# Supplementary material for: Vascular Remodeling in Moyamoya Angiopathy: From Peripheral Blood Mononuclear Cells to Endothelial Cells
Source: Int J Mol Sci. 2020 Aug 11;21(16):5763. doi: 10.3390/ijms21165763 (PMC7460840; doi:10.3390/ijms21165763)
Supplement: Supplementary file 1 [file ijms-21-05763-s001.zip › Supplementary Figure 1_Tinelli et al.docx]

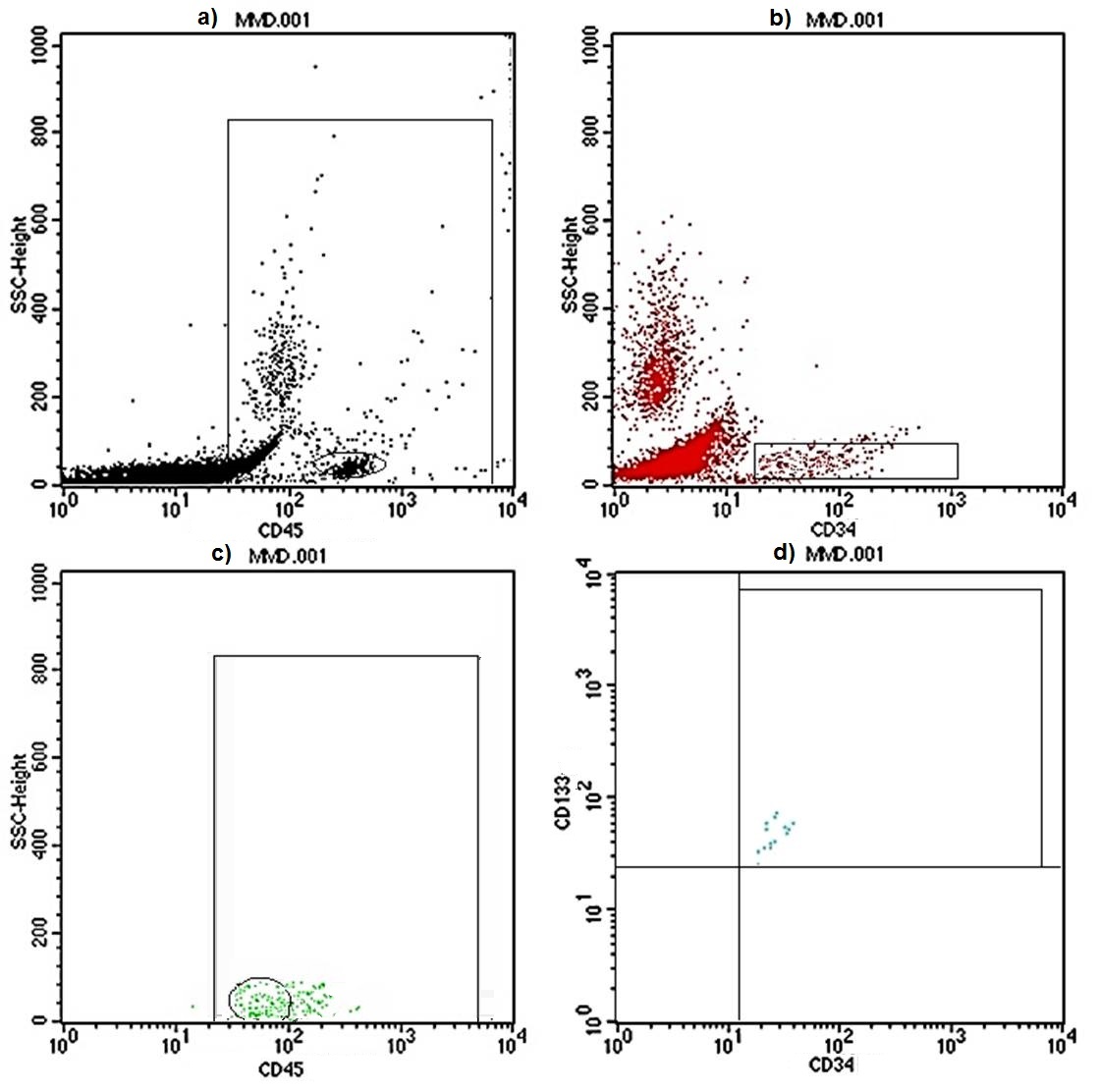


**Supplementary Figure 1**. **Flow cytometer analysis of circulating EPCs.** The EPC counts in the peripheral blood were determined by flow cytometry on whole blood samples: (A) CD45^+^ events on Gate1 region using VIO PRCP700-labeled antibodies against CD45; (B) proportion of CD34^+^ cells on CD45^+^-gated events was analyzed in the Gate2 region using FITC-labeled antibodies against CD34; (C) Gate3 region selected CD45^dim^ cells; (D) triple-positive cells were identified by the dual expression of CD34 and CD133 within the CD45^dim^-gated population. Since EPCs are rare in normal peripheral blood, at least 500 CD34^+^ events (B) per sample were acquired.
