# Supplementary material for: Vascular Remodeling in Moyamoya Angiopathy: From Peripheral Blood Mononuclear Cells to Endothelial Cells
Source: Int J Mol Sci. 2020 Aug 11;21(16):5763. doi: 10.3390/ijms21165763 (PMC7460840; doi:10.3390/ijms21165763)
Supplement: Supplementary file 1 [file ijms-21-05763-s001.zip › Supplementary Table 1_Tinelli et al.docx]

| **MA** | **Age,**  **gender** | **CVD type** | **NIHSS** | **U/B** | **Suzuki grading** | **Clinical features** | **Pharmacological therapy** |
| --- | --- | --- | --- | --- | --- | --- | --- |
| 1 | 44, f | HS | 5 | B | III | H, PSY | AE, AD |
| 2 | 58, m | HS | 6 | B | IV | PSY | AD |
| 3 | 37, f | HS | 0 | U | III | HT | AG |
| 4 | 47, f | IS | 8 | B | V | H, PI | AG, ST, AE |
| 5 | 42, f | IS | 2 | B | IV | HT | AG, ST |
| 6 | 46, f | Other | 0 | U | III | None | AG |
| 7 | 50, f | HS | 1 | B | IV | PSY | AD |
| 8 | 51, f | IS | 1 | B | IV | HoS, ET, DL, HHC | AG, ST, AE |
| 9 | 54, f | Other | 0 | U | III | PSY, HoS | na |
| 10 | 30, f | TIA | na | U | III | None | AG |
| 11 | 59, m | IS | 4 | B | IV | H, HoS | AG |
| 12 | 70, f | Other | 0 | U | na | H, DL, HoS | AG |
| 13 | 22, f | TIA | 10 | B | V | PSY, H | None |
| 14 | 30, m | Other | 0 | B | V | AA, DL | None |
| 15 | 63, m | HS | 0 | B | IV | H, DM, DL, IHH, HT | ST, CCB |
| 16 | 47, f | TIA | na | B | IV | PSY, H, HoS, PI | AG, AD |
| 17 | 52, f | Other | 0 | B | V | PI | None |
| 18 | 39, f | HS | 0 | B | na | DL, HoS, PI | ST |
| 19 | 45, f | IS | 0 | B | IV | PI, ET, HHC | AG, AHHC |
| 20 | 60, f | IS | na | B | V | H, DL | AG, ST |
| 21 | 52, f | HS | 0 | U | IV | None | None |
| 22 | 9, m | TIA | 2 | B | III | None | AG, AE |
| 23 | 5, m | IS | 6 | B | IV | None | AG, AE |
| 24 | 11, m | IS | 0 | U | III | None | AG, AE |
| 25 | 15, f | TIA | 0 | B | IV | None | AG |
| 26 | 16, f | Other | 0 | B | III | None | AG |
| 27 | 71, f | IS | 7 | B | na | DL | AG, ST |
| 28 | 49, m | IS | 4 | B | na | DM, HoS, DL | AG |
| 29 | 28, f | Other | 0 | U | IV | PI | AG |
| 30 | 55, f | TIA | 0 | B | III | H | AG, AH |
| 31 | 36, f | Other | na | U | VI | PI, ET, HT | AD |
| 32 | 56, f | IS | 0 | B | na | H, DL, HoS, HHC | AG, ST |
| 33 | 37, f | IS | 2 | B | VI | PSY, DL, H, ET, HT | AG, ST, AH |
| 34 | 45, m | na | na | B | VI | PSY, DL, HoS, AA, HT | AG, ST |
| 35 | 53, m | TIA | 0 | U | na | PSY, H, DL | AG, ST, AH, AD |
| 36 | 43, f | IS | 4 | B | III | None | AG, AE, ST |
| 37 | 16, f | IS | 0 | B | na | None | None |
| 38 | 56, f | IS | 8 | B | II | PSY, H, DL, IHH | AG, AE, ST, CCB |
| 39 | 20, m | IS | 2 | B | na | HT | AG, AE |
| 40 | 30, m | Other | 0 | U | na | AA, HoS, PI | None |
| 41 | 9, m | TIA | na | B | II | None | AG |
| 42 | 6, f | TIA | 0 | B | VI | None | AG |
| 43 | 42, m | Other | na | B | III | PSY | AG |
| 44 | 26, f | TIA | na | B | na | None | AG |
| 45 | 53, f | Other | na | B | III | H, DL | AG, ST |
| 46 | 11, m | None | na | U | II | None | AG |
| 47 | 30, f | IS | 5 | B | III | HoS | AG |

**Supplementary Table 1.** Demographic, clinical and neuroradiological features of 47 MA patients (AA, alcohol abuse; AD, antidepressant; AE, antiepileptic; AG, antiaggregants; AH, antihypertensive; AHHC, anti-hyperhomocysteinemia; B, bilateral; CCB, calcium channel blockers; CVD, cerebrovascular disease; DL, dyslipidemia; DM, diabetes mellitus; ET, estroprogestinic therapy; f, female; H, hypertension; HHC, hyperhomocysteinemia; HoS, history of smoking; HS, hemorrhagic stroke; HT, head trauma; IHH, ischemic heart disease; IS, ischemic stroke; m, male; na, not available; NIHSS, National Institute of Health Scale; PI, physical inactivity; PSY, psychiatric disorder; ST, statins; TIA, transient ischemic attack; U, unilateral).
