# Supplementary material for: Vascular Remodeling in Moyamoya Angiopathy: From Peripheral Blood Mononuclear Cells to Endothelial Cells
Source: Int J Mol Sci. 2020 Aug 11;21(16):5763. doi: 10.3390/ijms21165763 (PMC7460840; doi:10.3390/ijms21165763)
Supplement: Supplementary file 1 [file ijms-21-05763-s001.zip › Supplementary Table 2_Tinelli et al.docx]

|  | All patients (n = 47) | | Selected patients (n = 22) | |
| --- | --- | --- | --- | --- |
|  | Association with EPCs | | Association with EPCs | |
| Variables | Coef. (SD) | *p value* | Coef. (SD) | *p value* |
| Age (years) | -0.1354 (.1094) | *0.224* | -0.06197 (.0965) | *0.532* |
| Sex | -5.917 (4.033) | *0.151* | -5.412 (2.996) | *0.094* |
| Suzuki grading | 1.772 (1.147) | *0.121* | 3.018 (1.202) | *0.026** |
| CVD type | 1.980 (.9905) | *0.053* | 0.1085 (1.061) | *0.920* |
| Bilateral condition | -0.53819 (4.060) | *0.895* | 5.542 (2.522) | *0.047** |

**Supplementary Table 2.** Multivariate linear regression of EPC counts with regard to clinical characteristics of MA patients, considering both heterogeneous (n = 47) and homogeneous (n = 22) group. Multivariate regression analysis adjusted for age and sex was applied to assess the independence of disease severity features. EPCs levels were used as dependent variable for each of single feature. Tests were considered significant if the *p value* was <0.05. All analyses were performed using STATA 8.0 software (StataCorp LP, College Station, Texas, US) (CVD type, cerebrovascular disease type).
