# Supplementary material for: Vascular Remodeling in Moyamoya Angiopathy: From Peripheral Blood Mononuclear Cells to Endothelial Cells
Source: Int J Mol Sci. 2020 Aug 11;21(16):5763. doi: 10.3390/ijms21165763 (PMC7460840; doi:10.3390/ijms21165763)
Supplement: Supplementary file 1 [file ijms-21-05763-s001.zip › Supplementary Table 3_Tinelli et al.docx]

| Day 7 | | | | Day 17 | | |
| --- | --- | --- | --- | --- | --- | --- |
|  | HD | MA | *p value* | HD | MA | *p value* |
| n | 10 | 12 |  | 8 | 9 |  |
| VEGF-A | 22.86 ± 8.29 | 17.07 ± 9.23 | *0.297* | 5.85 ± 3.34 | 5.55 ± 3.94 | *0.918* |
| HGF | 1817 ± 1246.62 | 1306 ± 647.13 | *0.497* | 126.01 ± 178.18 | 340.34 ± 472.14 | *0.597* |
| TGF-β1 | 91.94 ± 116.75 | 161.75 ± 134.19 | *0.487* | 54 ± 62.92 | 32.75 ± 25.96 | *0.692* |
| CCL5/RANTES | 120.57 ± 77.78 | 59.62 ± 31.59 | *0.024* | 33.64 ± 26.52 | 27.41 ± 17.08 | *0.581* |
| CCL2/MCP-1 | 898.46 ± 412.9 | 777.83 ± 767.11 | *0.645* | 197.20 ± 422.84 | 135.76 ± 171.10 | *0.709* |
| IL8/CXCL8 | 45.30 ± 3.45 | 41.73 ± 6.67 | *0.125* | 33,47 ± 12,83 | 43,38 ± 11,30 | *0.115* |

**Supplementary Table 3.** VEGF-A, HGF, TGF-β1, CCL5/RANTES, CCL2/MCP-1 and IL-8/CXCL8 concentration (pg/ml) in conditioned media collected from EPC cultures at 7 and 17 days after seeding. Data were expressed as mean ± SD and statistical significance *(*p < 0.05*, ***p < 0.01*, ****p < 0.001*) was calculated through Student’s t-test. Values of at least three independent experiments are shown.
